# Digital phenotyping of migraine aura

**DOI:** 10.3389/fneur.2026.1967778

**Published:** 2026-10-22

**Authors:** Parisa Gazerani, Igor Petrušić, Marcos Fabio DosSantos

**Affiliations:** 1 Department of Life Sciences and Health, Faculty of Health Sciences, Oslo Metropolitan University, Oslo, Norway; 2 Advanced Health Intelligence and Brain-inspired Technologies (ADEPT), Faculty of Technology, Art and Design (TKD), Oslo Metropolitan University, Oslo, Norway; 3 Institute for Medical Research, University of Belgrade, Belgrade, Serbia; 4 Laboratório de Morfogênese Celular (LMC), Instituto de Ciências Biomédicas (ICB), Universidade Federal do Rio de Janeiro (UFRJ), Rio de Janeiro, Brazil; 5 Programa de Pós-Graduação em Neurociência Translacional (PGNET), Instituto de Ciências Biomédicas (ICB), Universidade Federal do Rio de Janeiro (UFRJ), Rio de Janeiro, Brazil; 6 Programa de Pós-Graduação em Medicina (Radiologia), Universidade Federal do Rio de Janeiro (UFRJ), Rio de Janeiro, Brazil; 7 Programa de Pós-Graduação em Medicina (Anatomia Patológica), Universidade Federal do Rio de Janeiro (UFRJ), Rio de Janeiro, Brazil

**Keywords:** cortical spreading depolarization, digital biomarkers, digital phenotyping, ecological momentary assessment, headache, migraine aura, precision neurology, wearable technologies

## Abstract

Migraine aura is a transient, focal neurological phenomenon, characterized by evolving visual, somatosensory, language, and, to a lesser extent, motor disturbances, among other symptoms that reflect dynamic alterations in brain function, involving not only cortical, but also subcortical structures. Despite advances in understanding its neurobiology, including the role of cortical spreading depolarization, migraine aura remains incompletely characterized. This is most likely because of its unpredictable onset, brief duration, individual and symptom variability, and reliance on retrospective clinical assessment. Consequently, its temporal evolution and biological heterogeneity remain to be fully elucidated. Digital phenotyping has emerged as a framework for characterizing health and disease through continuous real-world data collected using personal digital technologies. Here we examine the rationale for considering the potential application of digital phenotyping to migraine aura. We argue that aura’s unique biological and temporal characteristics can make it a suitable choice for continuous multidimensional assessment. Integrating patient-reported symptoms with physiological, behavioral, and environmental data may improve phenotypic resolution. Consequently, it may help advance mechanistic understanding and capture within-person variability. Therefore, we present a conceptual framework alongside the methodological and translational challenges of digital phenotyping, including implementation-related challenges. Rather than replacing established clinical and neurobiological approaches, digital phenotyping is presented as a complementary framework that, if rigorously validated, could potentially advance our understanding of aura and migraine aura research, support the institution and consolidation of precision headache medicine, and facilitate patient care.

## 1 Introduction

Migraine is a common and disabling neurological disorder that affects more than one billion people worldwide (1, 2). Its clinical presentation extends beyond headache to include a broad spectrum of neurological, somatosensory, autonomic, cognitive, and affective manifestations that vary substantially between individuals and across attacks (3). This clinical heterogeneity reflects the multifaceted neurobiology of migraine and presents an ongoing challenge for understanding disease mechanisms, improving patient stratification, and advancing precision-based approaches to care (4, 5).

Among the diverse manifestations of migraine, aura represents one of its most distinctive and biologically informative features (6). Traditionally defined as a transient focal neurological syndrome, aura most commonly consists of gradually evolving visual symptoms (7) but may also involve somatosensory, language, motor, and other disturbances (8–10), with neurobiological correlates (11–13). Although experienced by a subset of approximately one-third of individuals with migraine (12), aura has attracted considerable scientific interest because it provides a rare opportunity to relate evolving clinical symptoms to an established neurobiological process (14–17). Experimental, neurophysiological, and neuroimaging evidence has identified cortical spreading depolarization as the most likely biological substrate underlying migraine aura, establishing one of the clearest links between dynamic changes in brain activity and the temporal evolution of neurological symptoms (18–20).

Despite these advances, migraine aura remains incompletely understood. Clinical assessment continues to rely predominantly on retrospective symptom descriptions obtained after attacks have resolved, whereas mechanistic investigations are largely confined to experimental or imaging studies that capture only a limited number of spontaneous aura episodes (14, 21). Consequently, important aspects of aura, including its temporal evolution, inter- and intra-individual variability, relationship to physiological and behavioral changes, and interactions with environmental context, remain poorly characterized under real-world conditions. This gap limits both mechanistic understanding and the ability to capture the full complexity of aura as a dynamic neurobiological phenomenon (22, 23). Recent advances in digital health technologies (e.g., use of wearable sensors, ecological momentary assessment), coupled with the application of machine learning algorithms (24–26) seem to offer new opportunities to address some of these challenges.

Digital phenotyping (27), originally defined as the moment-by-moment quantification of the individual-level human phenotype *in situ* using data collected from personal digital devices, has evolved into a broader framework for integrating active and passive data streams to characterize health and disease in everyday life. Applied to migraine aura, this approach may open new potential means to move beyond static, retrospective symptom assessment toward continuous, multidimensional characterization of evolving neurological events. Importantly, the value of digital phenotyping lies not simply in the collection of more data, but in its potential to integrate subjective experiences with physiological, behavioral, and environmental information while preserving their temporal relationships. More broadly, digital phenotyping has recently been proposed as a transformative approach for migraine research and management, highlighting its potential to advance individualized phenotyping, digital biomarker discovery, attack prediction, and personalized care (28).

This Perspective examines the rationale for applying digital phenotyping to migraine aura and proposes a conceptual framework for its future development. It argues that migraine aura represents a unique model for continuous real-world phenotyping and critically discusses the opportunities, methodological challenges, and future directions for translating this approach into research and clinical practice.

## 2 Migraine aura

### 2.1 Definition and clinical spectrum of migraine aura

Migraine aura is defined as a transient focal neurological syndrome that precedes or accompanies migraine attacks and consists of fully reversible visual, somatosensory, language, motor, brainstem, or retinal symptoms that develop gradually over minutes and typically resolve within 1 h (29, 30).

The current diagnostic criteria established by the International Classification of Headache Disorders (ICHD-3) provide a standardized framework for recognizing aura based on its temporal evolution and characteristic symptom profile (31). Although these criteria have substantially improved diagnostic consistency, they were primarily developed for clinical classification rather than for capturing the evolving biological processes underlying aura (17, 29, 32).

Visual aura (33) is by far the most common presentation in patients with migraine with aura (14, 22, 23). Typical manifestations include scintillating scotomas, fortification spectra, photopsias, blurred vision, or transient visual field defects that gradually spread across the visual field (7). Somatosensory aura usually presents as unilateral paresthesia or numbness that, in most cases, begins in the hand and spreads to the arm, sometimes also affecting the ipsilateral tongue and face (12, 34–36). Language disturbances occur less frequently and are often accompanied by visual or somatosensory symptoms (14, 34). In that sense, perhaps aura should not be considered a single clinical entity but rather a heterogeneous spectrum of transient neurological disturbances. The symptom profile, sequence of progression, duration, and associated headache characteristics may differ substantially between individuals and even across attacks experienced by the same patient (9, 14). This variability highlights the complexity of aura and suggests that conventional symptom-based classification may only partially reflect its underlying biological diversity (21).

### 2.2 Epidemiology and clinical significance

Migraine with aura affects approximately 25–30% of individuals with migraine, although reported prevalence varies depending on the population studied, diagnostic methodology, and application of ICHD criteria (37–39). Aura may occur across all age groups, but typically begins during adolescence or early adulthood and can change in frequency, symptom profile, or even disappear over the course of the disease (34, 40–42). Migraine overall, and migraine with aura (43) specifically, is more common in women than men, reflecting the influence of biological sex and hormonal factors on migraine susceptibility and clinical expression (44, 45). Some studies report less frequent aura in women than in men, both during perimenstrual and non-perimenstrual migraine attacks (46).

Beyond its diagnostic importance, aura has significant clinical implications. Epidemiological studies consistently demonstrate that migraine with aura is associated with an approximately two-fold increased relative risk of ischemic stroke, particularly among younger women, smokers, and users of estrogen-containing contraceptives, although the absolute risk remains low (47–49). Migraine with aura has also been associated with differences in disease burden, cortical excitability, comorbidity profiles, and response to preventive therapies compared with migraine without aura, suggesting that aura represents more than simply an accompanying symptom of migraine (50, 51).

Consequently, identifying aura is not only essential for accurate diagnosis but also for improving patient stratification, understanding disease mechanisms, assessing vascular risk, and advancing individualized approaches to migraine management (6, 17, 35, 52, 53).

### 2.3 Aura as an evolving neurobiological process

One of the most distinctive features of migraine aura is its temporally structured evolution (6). Rather than appearing abruptly, symptoms typically develop gradually, propagate across functional domains, and resolve spontaneously over minutes (22, 54). This orderly progression closely mirrors the propagation characteristics of cortical spreading depolarization (CSD), a slowly propagating wave of neuronal and glial depolarization followed by prolonged suppression of cortical activity that is widely regarded as the principal neurobiological substrate of migraine aura (20, 55–57). Evidence from experimental studies, together with human neuroimaging demonstrating slowly propagating cortical hemodynamic changes during spontaneous aura, has substantially strengthened the link between CSD and the clinical manifestations of migraine aura (19, 58). However, the precise relationship between CSD and the diverse clinical manifestations of aura remains incompletely understood, particularly for non-visual aura and the considerable variability observed between patients (21, 59–61).

Moreover, aura extends beyond the subjective experience of neurological symptoms (62). As symptoms evolve, patients frequently modify their behavior by reducing physical activity, avoiding bright light or visually demanding environments, interrupting cognitive tasks, limiting social interactions, or seeking quiet surroundings. These behavioral adaptations occur alongside changes in sensory processing, cognition, autonomic regulation, and interaction with the surrounding environment (63–65). Therefore, aura should be viewed as a multidimensional neurobiological event that unfolds simultaneously across neurological, physiological, behavioral, and contextual domains rather than solely as a transient cortical phenomenon (36, 66, 67).

### 2.4 Why migraine aura represents a unique phenotype

Aura’s characteristics distinguish migraine aura from many other neurological phenomena. Aura is transient yet recurrent, clinically recognizable yet biologically informative, and characterized by a temporally organized evolution that patients are often able to describe with remarkable consistency (9, 53, 61). Unlike many neurological disorders in which disease progression is continuous or slowly evolving, migraine aura consists of discrete episodes that repeatedly occur within the same individual while exhibiting both reproducible patterns and clinically meaningful variability (9, 21, 68). It is worth mentioning that aura is not exclusive to migraine; similar visual phenomena have been described in epilepsy, especially in occipital lobe seizures, although the underlying mechanisms and clinical characteristics may differ (33, 69).

The combination of temporal organization, biological plausibility, and within-person reproducibility makes migraine aura an informative model for investigating transient changes in brain function. Individual attacks can be examined not only according to pain intensity, symptom type and duration but also in relation to symptom sequence, propagation, recovery, and accompanying physiological or behavioral adaptations. Such characteristics provide opportunities to investigate both stable individual traits and attack-to-attack variability, thereby offering insights into disease mechanisms that may not be apparent from cross-sectional clinical assessment alone (21, 70, 71). Despite these characteristics, clinical assessment remains largely dependent on retrospective symptom reporting and episodic evaluation. Consequently, important aspects of the temporal and multidimensional evolution of aura remain insufficiently characterized under real-world conditions. This disconnect between the biological behavior of migraine aura and its conventional characterization provides a rationale for exploring digital phenotyping as a complementary framework.

## 3 The measurement challenge: capturing a dynamic neurological phenomenon

Current diagnostic evaluation relies mainly on patient history, symptom recall, and established clinical criteria, all of which have substantially improved the recognition and classification of headaches (17, 31). However, these approaches primarily determine whether an aura has occurred rather than characterizing how it progressively unfolds over time. Consequently, much of the temporal complexity and multidimensional nature of aura remains inaccessible during routine clinical assessment. Neuroimaging studies have considerably expanded our understanding of migraine aura, particularly its underlying neurobiology and spatiotemporal propagation (29, 55, 71). A classical study, for example, demonstrated a progressively spreading BOLD signal through the occipital cortex accompanying visual scintillations, followed by reduced BOLD response corresponding to the perceived scotoma, providing direct evidence linking CSD to human visual aura (71). However, capturing aura as it naturally unfolds during everyday life remains a major challenge. Retrospective symptom reporting can oversimplify the assessment of a continuously evolving neurological event. Indeed, patients are often asked to reconstruct the onset, progression, duration, and sequence of symptoms after the headache attack has resolved, typically hours or days later. Although many individuals describe their aura with remarkable consistency, prospective studies have demonstrated considerable variability between attacks, while recall is also inherently influenced by bias (34, 53). Furthermore, clinical interviews less frequently capture behavioral adaptations, environmental influences, or concurrent physiological changes that accompany aura. Mechanistic approaches, including neuroimaging, electrophysiology, and experimental models, have substantially advanced our understanding of CSD and its relationship to aura evolution (19, 72–76). However, their application during spontaneous aura is constrained by its unpredictable onset, relatively short duration, and the practical limitations of clinic- and laboratory-based assessment.

We therefore observe a conceptual gap between biological understanding and the phenotypic characterization of an evolving, multidimensional phenomenon in which interactions occur among neurological symptoms, cortical activity, autonomic regulation, behavior, cognition, and environmental context. Currently, these domains are often investigated separately, using different methodologies and frequently in different study populations, leaving important questions about how these processes interact throughout the evolution of individual aura episodes and how they can be assessed in an integrated manner. We envision that digital phenotyping may bridge this gap and, rather than replacing these established methods, provide a complementary framework that integrates information across various domains involved in the experience of aura.

Another important challenge is capturing within-person variability. Although migraine aura often exhibits recognizable patterns within an individual, successive attacks may differ in symptom composition, propagation, duration, associated headache characteristics, and functional impact (53). Longitudinal characterization across repeated attacks may provide greater insight into phenotypic stability, intra-individual variability, and treatment response than isolated clinical observations. Prospective headache diaries, particularly electronic diaries, have already demonstrated the feasibility and value of longitudinal attack monitoring by reducing recall bias and capturing attack-specific characteristics over time (77–79). Ecological momentary assessment (EMA) further enables repeated assessment of symptoms, cognition, behavior, and context close to their occurrence and has been applied in migraine research, including the assessment of migraine-related cognitive burden (80). Digital phenotyping extends beyond these active self-report approaches by integrating time-stamped patient-reported information with passive or semi-passive physiological, behavioral, and contextual data collected during everyday life. Its proposed added value therefore lies not simply in digitizing symptom reporting, but in preserving and examining the temporal relationships among these complementary data streams as an aura episode evolves.

**Table** **1** translates the proposed four-domain framework into candidate measures, potential digital modalities, temporal sampling strategies, and outputs that could be evaluated in future prospective studies of migraine aura.

**Table 1:** Candidate measures for prospective digital phenotyping of migraine aura.

Domain	Candidate measure	Potential digital modality	Timing	Candidate output / Validation
Symptoms	Onset, symptom type, progression/propagation, duration, resolution	Smartphone-based event reporting, electronic diary/EMA, digital symptom mapping	Event-triggered at perceived aura onset and repeatedly through resolution	Time-stamped symptom trajectory; validate against prospective clinical characterization and reproducibility across attacks
Physiology	Heart rate/HRV, autonomic signals, sleep-related physiology; selected neurophysiological measures where feasible	Wearable sensors; research-grade physiological monitoring where applicable	Continuous baseline plus peri-aura and post-aura monitoring	Within-person deviation from baseline and temporal relationship to symptom evolution; assess analytical reliability and correspondence with established measures
Behavior	Physical activity, mobility, device interaction, interruption/resumption of usual activity	Wearable accelerometry and smartphone-derived behavioral measures	Continuous baseline and throughout aura/headache/recovery	Change from individualized baseline and temporal association with functional impact; assess reproducibility across repeated attacks
Context	Ambient light, sleep/wake context, stress or situational factors, environmental exposure where measurable	Smartphone/wearable sensors combined with brief contextual EMA	Baseline and peri-event, with time-stamped contextual annotation	Contextual conditions associated with aura onset/evolution; evaluate consistency within individuals and distinguish associations from causal or biological signals

EMA, ecological momentary assessment; HR, heart rate; HRV, heart rate variability. The candidate measures and modalities presented in this table are illustrative and are intended to guide future prospective investigation rather than to represent validated digital biomarkers of migraine aura. The information presented in this table is synthesized from the current literature (14, 28, 31, 70–72, 76) and is not intended to provide a systematic evidence summary.

For the research question considered here, a key unresolved limitation is the restricted temporal and multidimensional resolution with which individual aura episodes are usually observed. The hypothesis is that digital phenotyping may facilitate linking various dimensions of aura across time and in everyday settings as a complementary and still-developing framework.

## 4 Digital phenotyping: a framework for characterizing migraine aura

Digital phenotyping (81), such as smartphone digital phenotyping (82), has emerged as a methodological framework for characterizing health and disease through the continuous collection and analysis of data generated in everyday life, in real time, using non-invasive approaches with minimal (or in some cases, no) interference with physiological activities. The term was originally defined as the moment-by-moment quantification of the individual-level human phenotype *in situ* using data from personal digital devices (83–85). Since its introduction, digital phenotyping has evolved beyond smartphone-derived behavioral data to encompass multimodal information collected from smartphones, wearable sensors, and other connected technologies, integrating both active self-reports and passive sensor-derived data to characterize health and behavior in real-world settings, always with explicit informed consent from patients (28, 81, 86, 87). Therefore, digital phenotyping should not be regarded as a specific technology or individual device, but rather as a methodological framework for understanding human health through longitudinal, ecologically valid observations. Within this framework, active sensing refers to information intentionally provided by individuals, including symptom reports, electronic diaries, questionnaires, or EMA. In contrast, passive sensing involves the unobtrusive collection of physiological, behavioral, or environmental information through connected devices without requiring continuous user input, such as activity patterns, heart rate, sleep characteristics, ambient light exposure, or device interaction (83, 86, 87). The principal strength of digital phenotyping lies not simply in generating larger volumes of data but in preserving the temporal relationships among multiple dimensions of health as they evolve over time. This is particularly relevant for episodic neurological disorders, in which disease expression fluctuates and may not be adequately represented by isolated clinical assessments. Rather than providing single measurements, digital phenotyping enables repeated characterization of individual trajectories within their natural environment. Migraine aura has several characteristics that make it particularly well-suited to such an approach. Unlike many neurological phenomena, aura is transient, temporally organized, and perceptible to the individual experiencing it. Symptoms evolve gradually over minutes, often following recognizable patterns that patients can describe with considerable accuracy. At the same time, aura represents a biologically meaningful event generally accepted as linked to CSD, providing a rare opportunity to relate subjective symptom evolution to underlying neurobiological processes. Repeated aura episodes within the same individual further allow longitudinal investigation of within-person variability while preserving the ecological context in which attacks occur. Within this framework, the objective extends beyond the continuous recording of symptoms. Instead, digital phenotyping seeks to integrate subjective symptom evolution with objective physiological, behavioral, and contextual observations to generate a richer representation of the aura episode. Such multidimensional characterization may consequently help improve phenotypic resolution, facilitate investigation of disease mechanisms, and support more individualized approaches to research and care of affected individuals.

An important conceptual distinction should also be recognized here. Digital phenotyping describes the overall methodological framework rather than the measurements themselves. Raw sensor outputs are transformed into digital features, which may subsequently be combined and evaluated as candidate digital biomarkers if they demonstrate reproducible and clinically meaningful relationships with biological processes or clinical outcomes (88, 89). For migraine aura, however, validated digital biomarkers have not yet been established. Consequently, digital phenotyping should currently be viewed primarily as a research methodology capable of generating and testing hypotheses rather than as a clinically validated diagnostic or predictive tool. Future validation of candidate digital measures should therefore examine their temporal correspondence with prospectively reported aura evolution, reproducibility across repeated attacks, stability relative to individualized interictal baselines, and associations with established clinical or neurophysiological measures where feasible. Analytical validity, including measurement reliability and robustness across devices and settings, would also need to be established before clinical validity or utility could be evaluated.

## 5 A conceptual framework for dynamic phenotyping of migraine aura

Building on the current literature, we propose that migraine aura should be viewed as a dynamic neurobiological phenomenon whose complexity cannot be fully characterized through conventional clinical assessment alone. Accordingly, a complementary phenotypic framework is needed to integrate diverse sources of information into a coherent representation while preserving the temporal relationships among symptoms, physiology, behavior, and environmental context. We therefore position this four-domain framework as the central conceptual proposal of this Perspective. Within this framework, migraine aura should be characterized as an evolving phenotype rather than as a collection of isolated neurological symptoms. Aura unfolds through interactions among symptom evolution, physiological responses, behavioral adaptation, and environmental context. Capturing these dimensions together may provide a more complete representation of the aura episode than any single measurement in isolation. Consequently, we propose a conceptual framework comprising four complementary and interacting domains (**Figure** **1**). The first is the symptom dimension, reflecting the patient’s perceived symptom onset, progression, propagation, and resolution. The second is the physiological dimension, encompassing measurable biological responses, including autonomic and other physiological changes that accompany the evolving attack. The third is the behavioral dimension, describing changes in daily activities, functioning, movement, cognition, or adaptive behaviors that occur during an aura. The fourth is the contextual dimension, incorporating environmental and situational factors that may influence, accompany, or modify the aura experience. These domains were selected because they represent complementary aspects of aura that can realistically be repeatedly characterized during spontaneous attacks in everyday life and subsequently integrated into a unified phenotype, building on the general principles of digital phenotyping while extending them to migraine aura.

**Figure 1:**
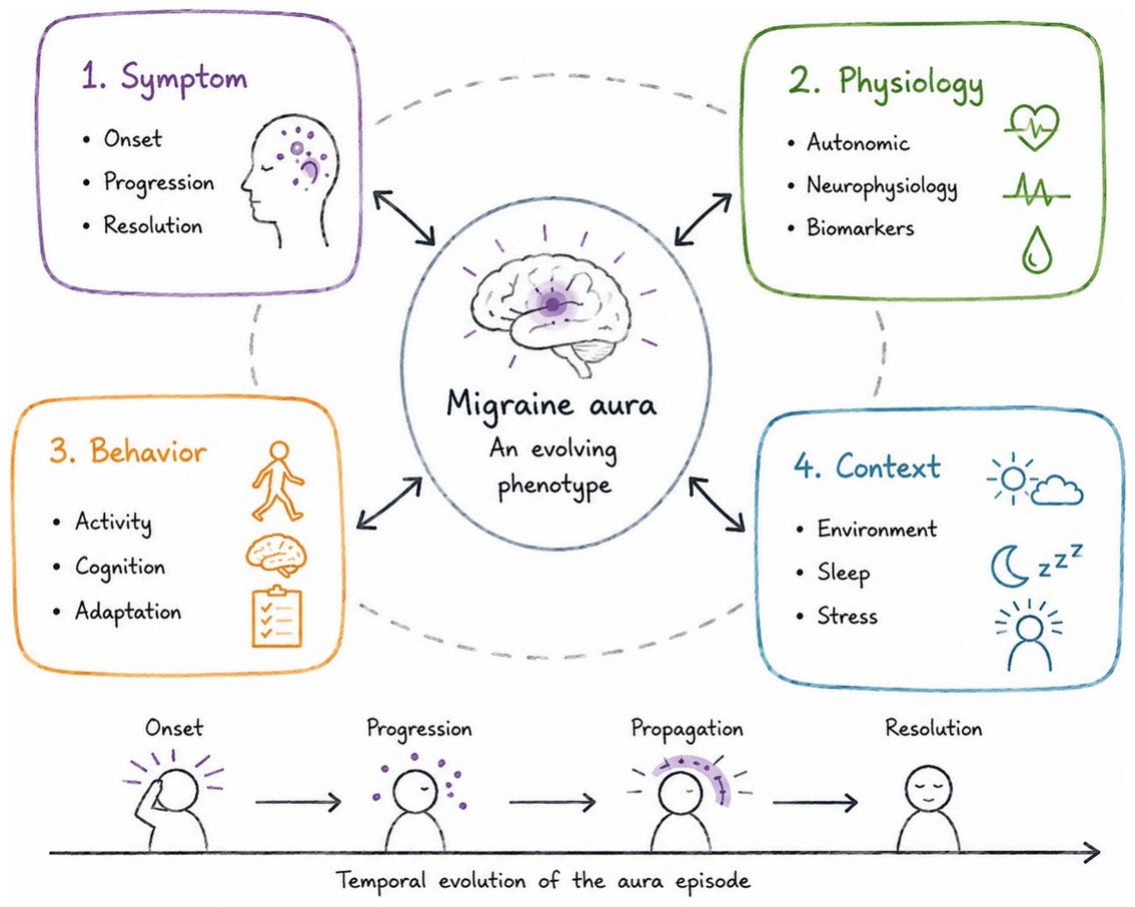
Conceptual framework for dynamic phenotyping of migraine aura. Migraine aura is conceptualized as an evolving neurobiological phenotype comprising four complementary and interacting domains: (1) symptom evolution, including onset, progression, propagation, and resolution; (2) physiological responses, encompassing measurable autonomic and other biological changes; (3) behavioral adaptation, reflecting alterations in activity, cognition, and function during the attack; and (4) contextual influences, including environmental and situational factors. These domains evolve throughout the temporal course of individual aura episodes and together provide a multidimensional representation of the aura experience. Digital phenotyping constitutes the methodological framework through which these complementary domains may be characterized longitudinally during spontaneous aura episodes using active self-reports (e.g., electronic diaries and ecological momentary assessment, EMA) together with passive or semi-passive data acquired from smartphones, wearable sensors, and other connected technologies. The framework illustrates how integrating these complementary data streams, while preserving their temporal relationships, has the potential to enhance conventional clinical assessment, especially when combined with deep-learning methods, and support a more comprehensive characterization of migraine aura.

### 5.1 Operationalizing the framework in prospective studies

A prospective digital phenotyping study of migraine aura could combine longitudinal baseline monitoring with event-triggered, time-resolved assessment of spontaneous aura episodes. An initial interictal monitoring period could establish individualized baselines for selected physiological, behavioral, and contextual measures. At perceived aura onset, an event-triggered protocol could initiate time-stamped symptom reporting and mapping of symptom progression, accompanied by intensified or annotated acquisition of wearable- and smartphone-derived measures across the relevant domains. Monitoring could continue through aura resolution and the subsequent headache and postdromal phases to characterize recovery and temporal relationships among data streams. Repeating this process across multiple attacks within the same individual would enable comparison with the interictal baseline and help distinguish reproducible within-person patterns from attack-specific variability. Such a design should be regarded as a prospective research model rather than a validated protocol, with the choice of measures, sampling frequency, monitoring duration, and event-triggering procedures requiring empirical evaluation.

Within this framework, patient-reported observations remain indispensable rather than being viewed as a limitation. Migraine aura is one of the few neurological phenomena in which patients are often able to recognize and describe symptom evolution with considerable temporal precision (34, 53). These observations therefore provide the temporal anchor against which physiological, behavioral, and contextual data can be interpreted. Importantly, the proposed framework is technology-agnostic. It is not tied to any specific wearable device, smartphone platform, sensor, or analytical algorithm. Instead, it is based on the principle of longitudinally integrating complementary sources of information acquired under real-world conditions to characterize the evolving phenotype of migraine aura. It is also worth mentioning that this framework does not assume that all domains contribute equally to every individual or every aura episode. Migraine is inherently heterogeneous, and the relative contribution of symptom, physiological, behavioral, and contextual dimensions is likely to vary both between individuals and across repeated attacks (53). Consequently, dynamic phenotyping should emphasize within-person longitudinal characterization rather than the identification of a single universal digital biomarker (87). Accordingly, the principal conceptual contribution of this perspective is to reposition migraine aura from a static clinical event to a dynamic, longitudinally evolving neurobiological phenotype that can be characterized through the integration of complementary domains over time while preserving their temporal relationships. This framework is intended to complement, rather than replace, any established clinical assessment and to provide a conceptual foundation for future mechanistic and translational research.

## 6 Opportunities for research and clinical translation

The proposed framework extends beyond improved documentation of migraine aura (90) by providing opportunities to investigate mechanisms, clinical phenotypes, and treatment response, and to support more individualized approaches to migraine research and care (28). Although many of these applications remain exploratory, they illustrate how longitudinal phenotyping may complement established experimental and clinical methodologies. One immediate opportunity lies in improving phenotypic resolution. Current classification systems distinguish aura according to symptom type and temporal criteria, which remain fundamental for diagnosis (31). However, they provide limited insight into how symptoms interact with physiological responses, behavioral adaptation, and environmental context throughout an individual episode. Integrating these complementary domains may reveal previously unrecognized patterns of aura expression, facilitate identification of clinically meaningful phenotypic subgroups, and improve patient stratification in both clinical practice and research (91).

Digital phenotyping may also advance mechanistic investigation. Although neuroimaging, electrophysiology, and experimental models have substantially improved understanding of CSD and aura signatures, they remain constrained by the difficulty of capturing spontaneous aura episodes outside the laboratory or clinic. Longitudinal observations collected during naturally occurring attacks may provide complementary information regarding the temporal relationships among symptom evolution, autonomic regulation, behavior, and environmental exposures. While such observations cannot directly measure CSD, they may generate testable hypotheses about the biological processes accompanying aura and inform the prioritization of future mechanistic studies. Taken together, these developments illustrate the potential of digital phenotyping to reshape the future pathophysiological characterization and clinical management of migraine with aura, an approach that can be further expanded to other contexts of both health and disease. It is worth noting that such advances must be implemented in accordance with rigorous ethical principles that evolve alongside technological progress, while always respecting individual autonomy and privacy.

A further opportunity lies in characterizing within-person variability. Migraine aura is often considered stereotyped, yet repeated attacks may differ in symptom composition, propagation, duration, associated headache characteristics, and functional consequences. Repeated longitudinal assessment may distinguish stable individual traits from attack-specific variation, thereby improving understanding of heterogeneity and potentially informing individualized management strategies.

This framework may also enhance therapeutic evaluation. Conventional outcome measures primarily assess headache frequency, pain intensity, or attack duration. Dynamic phenotyping offers the opportunity to evaluate treatment effects across multiple dimensions, including aura symptom evolution, physiological responses, functional performance, behavioral adaptation, and recovery. Such multidimensional outcomes may provide a more comprehensive assessment of pharmacological therapies, neuromodulation, and future digital interventions than traditional clinical endpoints alone.

More broadly, this approach aligns with the ongoing transition toward precision neurology and precision headache medicine (92, 93). Rather than relying exclusively on population-level averages, longitudinal phenotypic characterization may support individualized monitoring, improve participant selection for clinical trials, identify biologically relevant subgroups, and ultimately facilitate more personalized therapeutic decision-making. Importantly, these potential applications remain dependent on rigorous biological and clinical validation and should be regarded as future research priorities rather than established clinical practice. In this context, the V3 framework (Verification, Analytical Validation, and Clinical Validation) proposed by the Digital Medicine Society provides a useful foundation for evaluating digital measures, digital biomarkers, and related digital health technologies before clinical implementation (88). Accordingly, the opportunities outlined in this section should be interpreted as prospective directions for investigation rather than as currently established capabilities of digital phenotyping in migraine aura. Their realization will depend on addressing the scientific, methodological, and implementation challenges discussed below.

## 7 Challenges and future directions

While digital phenotyping offers opportunities for advancing migraine aura research, important scientific, methodological, and practical challenges must be addressed before broader implementation can be considered. Many of the concepts discussed in this Perspective remain hypothesis-generating and require rigorous prospective validation. Consequently, digital phenotyping should currently be regarded as an emerging research framework rather than an established clinical methodology (28). A fundamental challenge concerns biological validation. Digital features derived from smartphones, wearable sensors, and other connected technologies represent indirect measures of physiological and behavioral processes rather than direct measurements of CSD or other mechanisms underlying migraine aura. Demonstrating meaningful relationships between digital features and established neurobiological processes will require carefully designed studies integrating digital phenotyping with conventional clinical assessment, neuroimaging, electrophysiology, and complementary methodologies. Until such evidence is available, digital features should not be interpreted as proposed surrogates of CSD. Moreover, changes in measures such as physical activity, heart rate, device interaction, or ambient light exposure may reflect behavioral adaptation, headache-related pain, anxiety, medication use, environmental circumstances, or other contextual influences rather than aura-specific biological processes. Temporal association with an aura episode is therefore insufficient to establish biological specificity. Distinguishing candidate biological signals from behavioral consequences or contextual correlates will require comparison with individualized baselines, repeated observations across attacks, multimodal assessment, and validation against established clinical or neurophysiological measures where feasible.

The marked heterogeneity of migraine aura presents an additional challenge. Considerable variation exists in symptom profile, propagation, duration, frequency, and associated headache characteristics, both between individuals and across repeated attacks within the same individual (3, 21). This variability complicates the identification of reproducible digital signatures and suggests that individualized longitudinal characterization may ultimately prove more informative than attempts to identify a universal digital biomarker. Future studies should therefore consider both population-level patterns and within-person trajectories.

Practical implementation also requires careful consideration. Aura episodes are unpredictable, relatively brief, and to some extent infrequent, making prospective capture inherently challenging. Long-term monitoring may therefore be necessary to capture sufficient attacks, raising concerns about participant adherence, device usability, data completeness, and study feasibility. In addition, the diversity of digital platforms, wearable technologies, and analytical approaches highlights the need for standardized acquisition protocols, harmonized data-processing pipelines, transparent reporting standards, and independent validation to ensure reproducibility across studies.

Ethical, regulatory, and governance considerations will be equally important, particularly as artificial intelligence (AI) and related technologies become increasingly integrated into digital phenotyping frameworks (94). Emerging work on AI-enabled migraine care and digital twins highlights both the considerable potential of these approaches and the ethical, methodological, and regulatory challenges accompanying their implementation (95, 96). Continuous collection of physiological, behavioral, and contextual information inevitably involves sensitive personal data. Robust frameworks addressing informed consent, privacy, cybersecurity, data governance, algorithmic transparency, fairness, and equitable access should accompany technological development from the earliest stages of research. Responsible implementation will be essential to maintain public trust and ensure that future clinical applications remain patient-centered.

Ultimately, successful clinical translation will also depend on demonstrating clear added value beyond existing approaches. Digital phenotyping should complement rather than replace established diagnostic criteria, clinical evaluation, and mechanistic investigation. Its value will depend not on the volume of data collected but on its ability to generate clinically meaningful information that improves phenotypic characterization, advances understanding of migraine aura, and supports more informed clinical decision-making. Future research should therefore prioritize prospective longitudinal studies, multimodal validation against established biological and clinical measures, standardized analytical frameworks, and interdisciplinary collaboration among clinicians, neuroscientists, engineers, data scientists, and patients. Such efforts will determine whether digital phenotyping can evolve from a promising research methodology into a clinically useful approach for characterizing migraine aura and supporting future precision headache medicine.

## 8 Conclusion

Migraine aura remains one of the distinctive and biologically informative manifestations of migraine, yet its evolving nature continues to challenge conventional approaches to clinical assessment and mechanistic investigation. Although considerable progress has been made in understanding the neurobiology of aura, important aspects of its temporal evolution, biological heterogeneity, and interaction with physiological, behavioral, and environmental processes remain incompletely characterized. This Perspective argues that these challenges are fundamentally challenges of phenotyping. Accordingly, migraine aura should be regarded as an evolving neurobiological phenotype rather than a static clinical event. Within this framework, digital phenotyping is presented not as a technological solution, but as a methodological approach for integrating complementary dimensions of the aura experience over time, thereby enabling a more comprehensive characterization of spontaneous aura episodes under real-world conditions. Moreover, the potential of digital phenotyping should be interpreted with appropriate scientific caution. Robust biological validation, standardized methodologies, reproducible analytical frameworks, and clinically meaningful outcome measures remain essential before routine clinical implementation can be considered. Future progress will depend on interdisciplinary collaboration spanning neuroscience, headache medicine, digital health, engineering, and data science (97).

If prospectively validated, digital phenotyping has the potential to complement existing approaches by improving phenotypic resolution, generating new mechanistic hypotheses, and supporting more individualized approaches to migraine research and clinical care. To move toward this goal, future research should prioritize: (1) prospective, time-resolved characterization of spontaneous aura using standardized approaches; (2) validation of candidate digital measures against individualized interictal baselines, repeated attacks, and established clinical or neurophysiological measures where feasible; (3) assessment of within-person reproducibility and between-person heterogeneity; and (4) demonstration of whether multimodal digital phenotyping provides meaningful information beyond prospective electronic diaries or EMA alone. Addressing these priorities will be essential for determining whether digital phenotyping can progress from a promising research framework toward a reproducible and clinically meaningful approach to studying migraine aura.

## Data Availability

The original contributions presented in the study are included in the article/supplementary material, further inquiries can be directed to the corresponding author/s.
